# An Infinitely Expandable Cloning Strategy plus Repeat-Proof PCR for Working with Multiple shRNA

**DOI:** 10.1371/journal.pone.0003827

**Published:** 2008-11-27

**Authors:** Glen John Mcintyre, Jennifer Lynne Groneman, Anna Tran, Tanya Lynn Applegate

**Affiliations:** Johnson and Johnson Research Pty Ltd, Australian Technology Park, Eveleigh, New South Wales, Australia; East Carolina University, United States of America

## Abstract

Vector construction with restriction enzymes (REs) typically involves the ligation of a digested donor fragment (insert) to a reciprocally digested recipient fragment (vector backbone). Creating a suitable cloning plan becomes increasingly difficult for complex strategies requiring repeated insertions such as constructing multiple short hairpin RNA (shRNA) expression vectors for RNA interference (RNAi) studies. The problem lies in the reduced availability of suitable RE recognition sites with an increasing number of cloning events and or vector size. This report details a technically simple, directional cloning solution using REs with compatible cohesive ends that are repeatedly destroyed and simultaneously re-introduced with each round of cloning. Donor fragments can be made by PCR or sub-cloned from pre-existing vectors and inserted ad infinitum in any combination. The design incorporates several cloning cores in order to be compatible with as many donor sequences as possible. We show that joining sub-combinations made in parallel is more time-efficient than sequential construction (of one cassette at a time) for any combination of 4 or more insertions. Screening for the successful construction of combinations using Taq polymerase based PCR became increasingly difficult with increasing number of repeated sequence elements. A Pfu polymerase based PCR was developed and successfully used to amplify combinations of up to eleven consecutive hairpin expression cassettes. The identified PCR conditions can be beneficial to others working with multiple shRNA or other repeated sequences, and the infinitely expandable cloning strategy serves as a general solution applicable to many cloning scenarios.

## Introduction

Vector construction using REs is a fundamental procedure in modern molecular biology. A typical cloning strategy using REs involves the ligation of a digested donor fragment to a reciprocally digested recipient fragment. Vectors are built with a cluster of adjacent recognition sites known as a multiple cloning site (MCS) or polylinker, allowing the user to pick the most suitable enzyme(s). Despite the wide choice of REs available, typically only a subset of these are suitable in any given project. Suitability can be determined by ease of use, compatibility with other enzymes, but most commonly by the number of recognition sites present. Ideal cloning strategies contain only unique restriction sites (only present once) to ensure that cloning is directional and straightforward, hence requiring two unique sites for each insertion event. A single or blunt-ended site(s) can also be used but this is non-directional, inefficient and requires an increased screening effort.

As the vector size increases, the number of unique restriction sites common to both recipient and donor fragments decreases. This is typically not a problem in simple projects using recipient vectors up to several thousand bases (kb) long. However, creating a suitable cloning plan becomes increasingly difficult in complex strategies requiring repeated insertions and or large recipient vectors such as in constructing multiple shRNA expression vectors for RNAi studies. In some cases it may even be impossible to formulate an ideal construction plan for repeated insertions. With an increasing number of multiple shRNA studies using hairpins in ever-greater combinations (of 2 [1], [2], 3 [3], [4], 4 [5], [6], and 6 [7]), there is an increasing need for a universal solution with the capacity for unlimited expansion. There is also a need for a specialized PCR screening method that is capable of amplifying templates containing multiple repeated sequences, as we and others have found standard Taq reactions unsuitable [8]. To address these needs, a directional and infinitely expandable cloning strategy was devised based on ‘recycling’ several sets of unique recognition sites with compatible cohesive ends. We also developed a Pfu polymerase based PCR for amplifying multiple hairpin templates. Both the cloning strategy and PCR were verified by constructing plasmids with up to 11 individual cassettes by sequentially inserting donor fragments generated both by PCR and by excision from pre-constructed plasmids.

## Results

### Conceiving the cloning strategy

**Figure 1:**
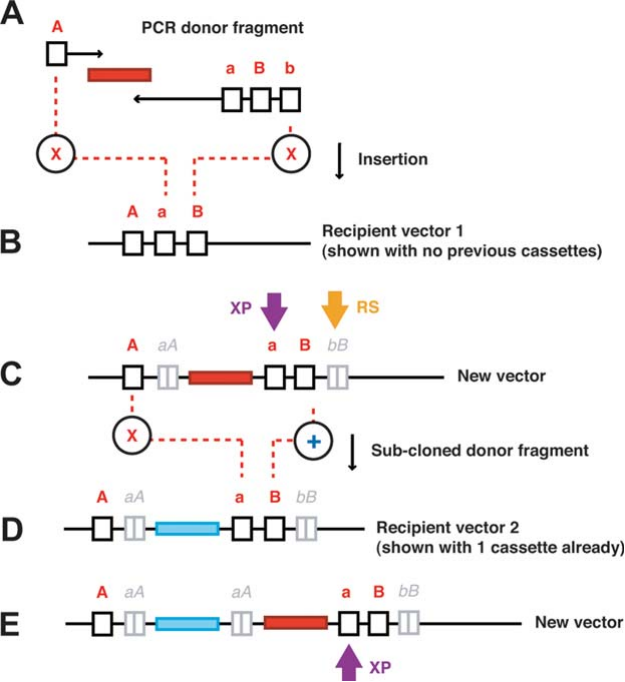
An infinitely expandable cloning strategy. The PCR generated donor fragment (A) is digested with ‘A’ and ‘b’ enzymes and ligated to the recipient vector (B) opened up with ‘a’ and ‘B’ enzymes destroying the original ‘A’, ‘b’, ‘a’, and ‘B’ sites in the process. The newly created vector (C) has the ‘a’ and ‘B’ sites reconstituted. Further insertions stack after each at the expansion point (XP), and each insertion leaves two non-functional digestion or ligation remnants, the downstream ones stacking together at a single point (RS). Sub-cloned donor fragments (C) from previously constructed vectors are excised with ‘A’ and ‘B’ enzymes and ligated to a recipient vector (D) opened up with the ‘a’ and ‘B’ sites. In this example the recipient vector (2) already has one inserted cassette, thus making a new vector with a total of two cassettes (E).

The cloning strategy that we devised was based on ‘recycling’ unique RE recognition sites through repeated destruction and replacement with every insertion event (**Figure 1**). The MCS in this design contains at least three recognition sites, designated ‘A’, ‘a’ and ‘B’; in that order. The PCR primers used to amplify donor inserts are made so that the forward primer introduces an ‘A’ recognition site and the reverse primer introduces ‘a’, ‘B’ and ‘b’ recognition sites (in that order). The sites need to be chosen carefully so that A:a and B:b have compatible cohesive ends, yet all sites are destroyed upon ligation. Digesting the PCR generated donor fragment with ‘A’ and ‘b’ enzymes, and ligating to the recipient vector opened up with ‘a’ and ‘B’ enzymes, creates a nascent vector where each of the original ‘A’, ‘b’, ‘a’, and ‘B’ sites are destroyed. New ‘a’ and ‘B’ sites are introduced into the nascent vector via the reverse primer. Thus ‘a’ and ‘B’ remain unique in the nascent vector and are positioned immediately 3′ to the last inserted donor fragment. The region from ‘A’ to ‘a’ in each vector is the ‘expansion point’ (XP); the point where each newly inserted donor is placed 3′ to the previous insertion and 5′ to the reconstituted cloning region. The non-functional remains of the ligated cloning sites, a|A and b|B, flank each PCR insertion, with the b|B remnants from all insertions stacking together 3′ of the reconstituted ‘B’ position.

After one or more PCR donor fragments have been inserted into the MCS, they can be similarly sub-cloned into a second vector that does or does not already contain inserted donor fragments. Sub-cloning vector-derived fragments requires the donor fragment to be excised from the first vector with ‘A’ and ‘B’ enzymes. The second, or recipient vector is opened up with the ‘a’ and ‘B’ sites. Upon ligation the ‘A’ and ‘a’ sites are destroyed, but a new ‘A’ is introduced into the nascent vector via the donor fragment. The ‘B’ site is maintained on ligation and only present once in the nascent vector. Therefore, the nascent vector has the sub-cloned donor fragment positioned at the same expansion point at which PCR generated donor fragments would be inserted. As before, the reconstituted cloning sites remain unique and indistinguishable in layout and functionality from those in the original recipient vector. Unlike PCR insertions, sub-cloned donor fragments leave only one set of recognition site remnants; a|A (non-functional) positioned 5′ to the current insertion.

### Selecting the recognition sites

Of the 76 enzymes with compatible cohesive ends (NEB catalog & Technical Reference, 2007–08), only 4 pairs were identified that were suitable for the recipient plasmid used herein (a 7 kb carrier plasmid encoding a lentiviral transfer vector), enabling the construction of two different cloning sets. The 8 enzymes were divided into two ‘core’ sets such that the enzymes with the most similar buffer requirements were grouped together (**Table 1**). The core 1 set (c1) was composed of *Spe* I (exchangeable for *Xba* I) and *Nhe* I, as well as *Bsr* GI and *Bsi* WI. The core 2 set (c2) was composed of *Mlu* I and *Asc* I, as well as *Pac* I and *Asi* SI. The dual core design allows for maximum flexibility and compatibility with donor sequences that may include core sites.

**Table 1:** Selected RE recognition sites with compatible cohesive ends.

				NEB Buffer activity (%)				
Usage	Enzyme	Site	Compatible	1	2	3	4	PCR
c1 MCS	*Nhe* I	G|CTAGC	*Spe* I / *Xba* I	100	100	10	**100**	100
c1 MCS, c1 sub	*Bsr* GI	T|GTACA	*Bsi* WI	25	**100**	10	**100**	<25
c1 PCR, c1 sub	*Spe* I	A|CTAGT	*Nhe* I / *Xba* I	**75**	**100**	25	75	**100**
c1 PCR	* ***Bsi*** ** WI**	C|GTACG	*Bsr* GI	**100**	**100**	100	25	**50**
c2 MCS	*Asc* I	GG|CGCGCC	*Mlu* I	0	10	10	**100**	100
c2 MCS, c2 sub	*Pac* I	TTAAT|TAA	*Asi* SI	100	**75**	10	**100**	100
c2 PCR, c2 sub	*Mlu* I	A|CGGCT	*Asc* I	25	**75**	**100**	50	**50**
c2 PCR	*Asi* SI	GCGAT|CGC	*Pac* I	50	100	**100**	50	**100**

Four pairs of RE recognition sites with compatible cohesive ends were suitable for the plasmid used in this study. The 4 pairs were divided into 2 ‘core’ sets so that the enzymes with the most similar buffer requirements were grouped together, based on the % activity of each enzyme in the 4 different New England Biolabs buffers plus standard PCR buffer (catalog & Technical Reference, 2007–08). ^*^ *Bsi* WI was optimally active at 55°C and 50 % active at 37°C.

### Assembling the cores and primers

**Figure 2:**
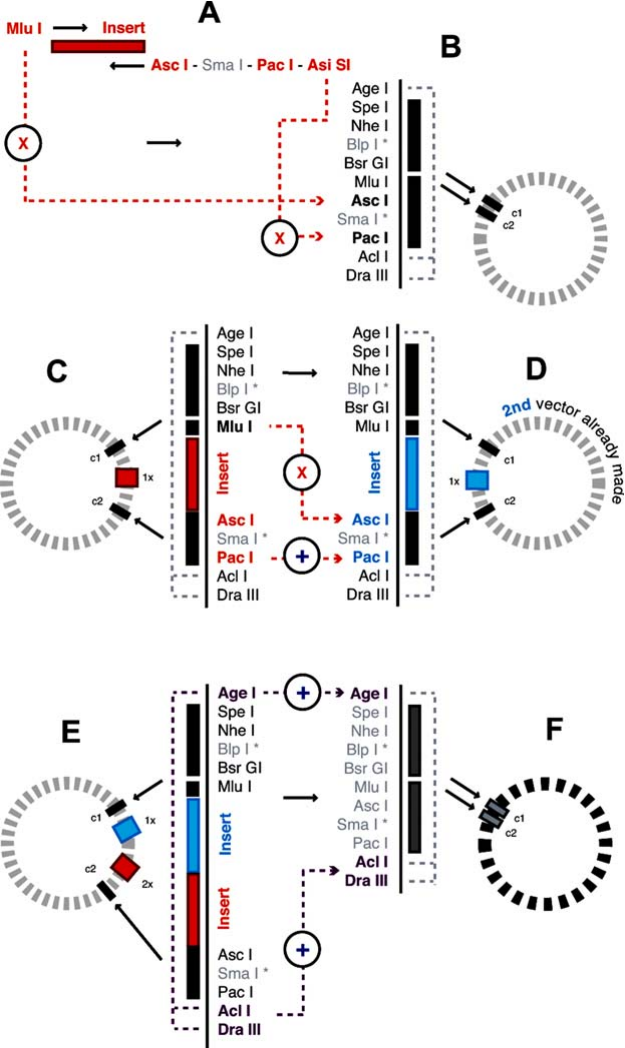
Site allocation and compatibilities. The MCS inserted into the recipient plasmid was assembled from *Age* I, *Spe* I, *Nhe* I, *Blp* I, *Bsr* GI, *Mlu* I, *Asc* I, *Sma* I, *Pac* I, *Acl* I, and *Dra* III. PCR donor fragments made with core 2 primers (A) are digested with *Mlu* I and *Asi* SI and inserted into *Asc* I and *Pac* I (B). Previous insertions (C) are excised with *Mlu* I and *Pac* I and sub-cloned into another vector prepared either previously or in parallel with (C) and opened with *Asc* I and *Pac* I (D). Entire cloning regions (all cores and inserts) can be shuttled between vectors (E–F) using the external shuttling sites, *Age* I and *Acl* I or *Dra* III. * *Blp* I and *Sma* I were included as spacers to distance the two sites to be double digested for receiving inserts.

The allocation and placement of each site in the MCS and or PCR primers was based on compatibility with the recipient plasmid (e.g. *Spe* I and *Mlu* I were both present in the recipient plasmid outside of the intended MCS) and further maximizing double digestion compatibilities within each set. The sites chosen for the MCS were *Spe* I (equivalent to the conceptual ‘A’ site), *Nhe* I (‘a’) and *Bsr* GI (‘B’) from the core 1 set, and *Mlu* I (‘A’), *Asc* I (‘a’) and *Pac* I (‘B’) from the core 2 set (**Figure 2**). An additional (unique) site was placed between the two sites within each pair, acting as a small spacer to facilitate the double digestion of sites in close proximity to one another. It also built-in a further option for uniquely digesting the vector(s) within each core set if required. The insertion sites included in the core 1 PCR primers were *Spe* I (‘A’) in the forward primer, and *Bsi* WI (‘b’) in the reverse primer. The sites chosen for the core 2 PCR primers were *Mlu* I (‘A’) in the forward primer, and *Asi* SI (‘b’) in the reverse primer. The MCS also included several other unique recognition sites flanking the entire cloning region; *Age* I at the 5′ end and *Acl* I and *Dra* III at the 3′ end. This provided extra cloning points to directionally expand the 3′ end and added an external shuttling capacity to simultaneously move both cores (plus inserts) between different vectors containing the same MCS. Using external sites for shuttling added another level of tolerance for donor sequences that contained core sites. Hypothetical construction scenarios testing repeated PCR and sub-cloned insertions in both cores were successfully simulated using Vector NTI (v.10.3.0, 2006) (Invitrogen).

### Repeated insertions using PCR generated donor fragments

**Figure 3:**
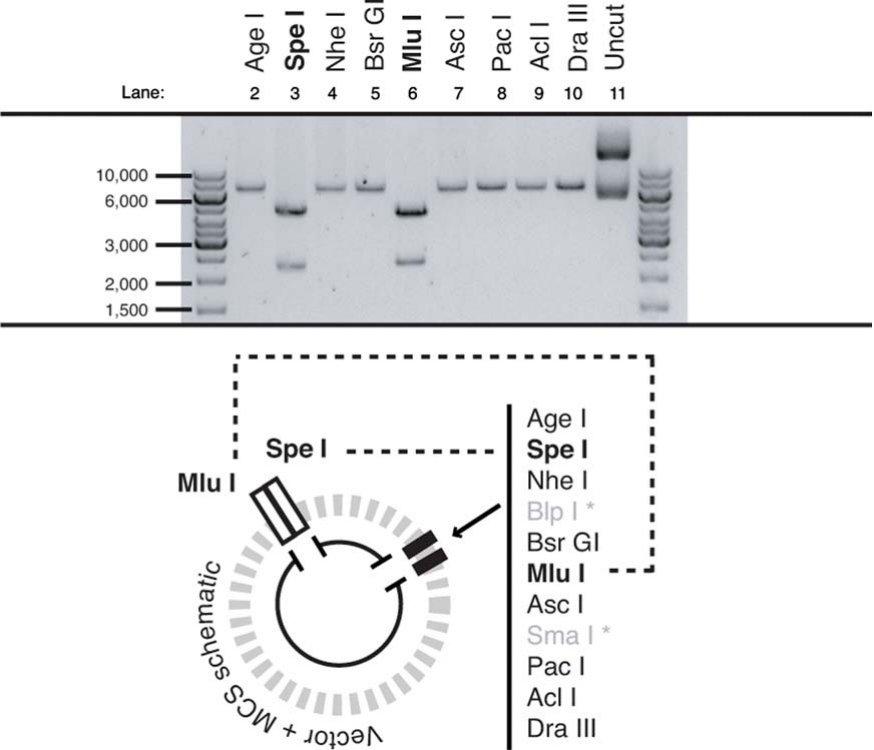
MCS functionality in the recipient plasmid. The recipient plasmid modified by insertion of the designed MCS was digested individually with *Age* I, *Spe* I, *Nhe* I, *Bsr* GI, *Mlu* I, *Asc* I, *Pac* I, *Acl* I, and *Dra* III to confirm their usability. All digestions linearized the plasmid, except for *Spe* I and *Mlu* I that were also present in the plasmid backbone and thus gave an expected two fragments (this did not impact on their role in the cloning strategy).

**Figure 4:**
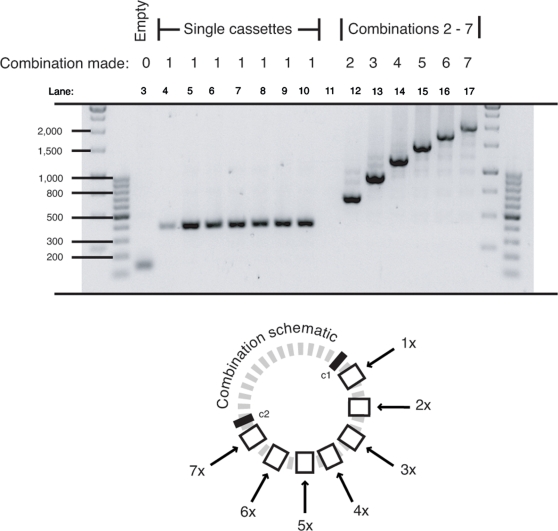
Combinations up to seven built from PCR donor fragments. Seven different hairpin expression cassettes were amplified with the core 2 primers and inserted both individually and sequentially to create combinations of increasing number up to seven in the recipient plasmid. Insertions were confirmed by PCR screening using primers that flanked the MCS region. Each insertion added ∼270 bp to the size of the previous recipient plasmid.

The core 2 enzymes were selected to demonstrate the practical feasibility of the strategy. The project for which this cloning strategy was devised required the insertion of at least 6 hairpin expression cassettes (approximately 270 bp each) into a single recipient plasmid. Each unique hairpin was already present in a common plasmid backbone (under the control of the human H1 promoter). Seven PCR donor fragments were prepared with common core 2 PCR primers as each cassette shared identical flanking sequence (enabling the same primers to be used in all cases). The recipient plasmid was first modified by insertion of a synthetic DNA fragment containing the designed MCS. The MCS sequence was confirmed by automated sequencing and tested by RE digestion (**Figure 3**). The modified plasmid was then opened with the core 2 recipient enzymes, *Asc* I and *Pac* I, creating a suitable recipient fragment. The donor fragments were digested with the core 2 donor enzymes *Mlu* I and *Asi* SI, and a single fragment was inserted into the recipient plasmid. Successful construction was demonstrated by amplification of the MCS (plus insert) region (**Figure 4**). Once selected and prepared, the nascent plasmid (now containing one cassette) was digested with the core 2 recipient enzymes, *Asc* I and *Pac* I, and ligated to a second PCR donor fragment. This process was repeated successfully for up to 7 expression cassettes as shown by Pfu-based PCR analysis; the development of which is described in the following section.

### A Pfu-based PCR to better amplify repeated sequences

**Figure 5:**
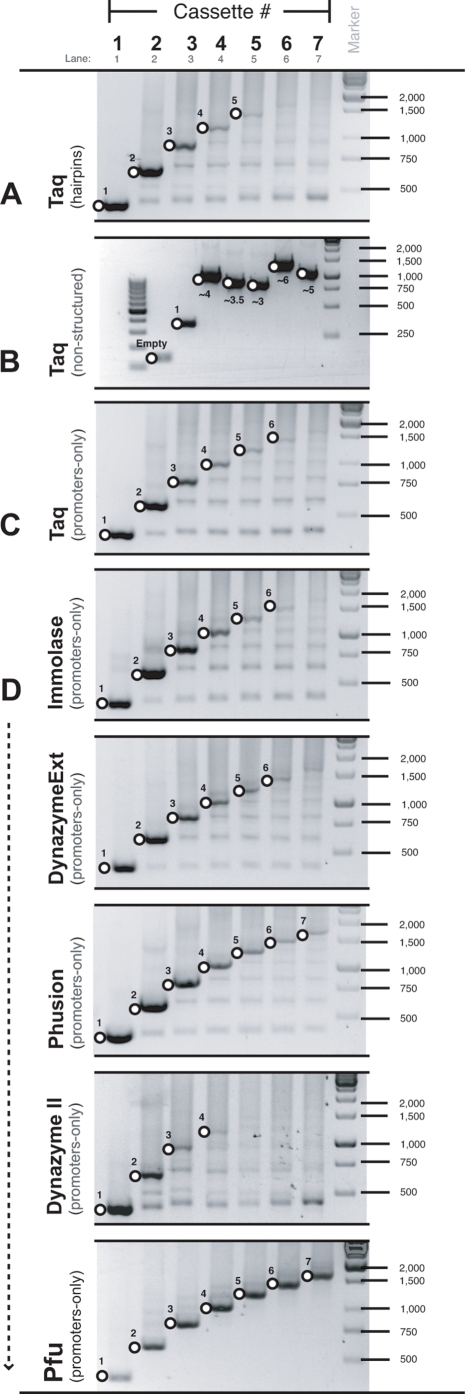
Pfu was used to efficiently amplify repeated sequences by PCR. (A) Taq-based PCR was unsuitable for screening multiple insertions as it produced strong intermediate-sized products and weak specific-products when amplifying templates containing 1 to 7 hairpin expression cassettes. (B) A series of approximately equivalent sized plasmids with non-structured (& non-repeated) inserts was successfully amplified with Taq. (C) Taq was unsuitable for amplifying templates containing 1 to 7 promoter-only cassettes (no hairpin sequences). (D) Several different polymerases (Phusion, Dynazyme EXT, Dynazyme II, Immolase and Pfu) were tested with the promoter-only series of vectors using the manufacturers recommended starting conditions.

While Taq-based PCR was suitable for generating individual PCR donor fragments, it proved to be unsuitable for screening multiple insertions as it produced strong intermediate-sized products and weak specific-products (**Figure 5a**). Moreover, it became ineffective for screening combinations containing 5 or more repeated expression cassettes. To show that poor product formation was not due to the large amplicon size (irrespective of repeated sequence), a series of non-structured plasmids was built of similar sizes to the multiple hairpin plasmids made. All were successfully amplified with standard Taq conditions (**Figure 5b**). Another series of control plasmids was built that also had up to 7 repeated cassettes each of which contained an identical promoter, but no shRNA. Like the multiple hairpin plasmids, it was also difficult to generate full-length products from these vectors (**Figure 5c**). Several different polymerases (Phusion, Dynazyme EXT, Dynazyme II, Immolase and Pfu) were tested with this series of vectors using the manufacturers recommended starting conditions (**Figure 5d**). Of these, Pfu was clearly the best. The optimal conditions for Pfu (with the plasmid and screening primers used here) was determined by testing combinations of cassette number, MgCl_2_ concentration, DMSO addition and annealing temperature. Optimal conditions included 5 % DMSO and a total MgCl_2_ concentration of 3.5 mM. Multiple cassettes were most efficiently amplified using Pfu with an annealing temperature of 66°C, more than 15°C higher than suggested by the manufacturer. Interestingly, short, non-repeated sequences (i.e. single expression cassettes) were better amplified using Pfu at a lower annealing temperature of 55–61°C, or with Taq under standard conditions.

### The time-saving benefits of sub-cloning

**Figure 6:**
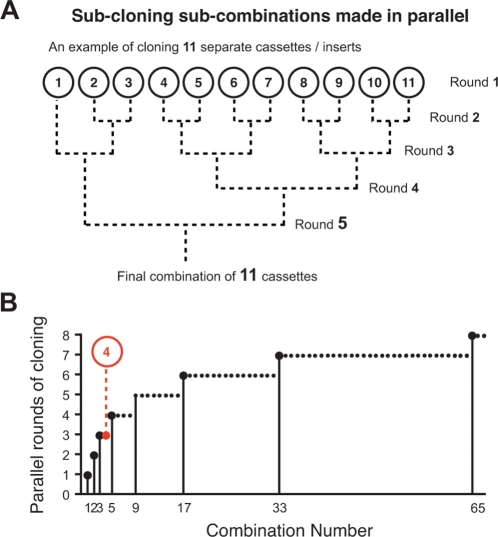
Sub-cloning sub-combinations is the most time-efficient way to build any combination greater than four. (A) The sub-cloning protocol can hasten the completion of large projects through parallel lines of construction in multiple vectors that are progressively joined together. For example, a combination of 11 could be made in 11 consecutive rounds of sequential PCR insertions, or more (time) efficiently in 5 rounds of sub-cloning sub-combinations. (B) The minimal number of rounds required to complete any given combination (n) was found by: ⌈log_2_(n)⌉+1 (when starting from scratch). Calculations show that sub-cloning is the most time-efficient construction strategy for any combination of four or more insertions (solutions shown for all combinations up to 65).

Inserting each PCR generated fragment one at a time into the destination vector is straightforward and ideal for small projects with few insertions. However, large projects can take a long time to complete as each sequential ‘round’ of cloning can take anywhere from several days to a week due to the time taken for bacterial growth. The sub-cloning protocol can hasten the completion of large projects through parallel lines of construction in multiple vectors that are progressively joined together until the final combination is attained. This is because several vectors can be made simultaneously in almost the same amount of time per ‘round’ of cloning as taken for one. For example, a combination of 11 could most efficiently be assembled in 5 rounds of cloning (**Figure 6a**). This is done by making 11 individual vectors in the first round, then joining these into 5 sub-combinations of 2 in the second round (plus 1 remainder), 2 sub-combinations of 4 and 1 sub-combination of 3 in the third, 1 sub-combination of 8 in the fourth (plus the sub-combination of 3 as a remainder), and finally connecting the sub-combinations of 8 and 3 together in the fifth. Assuming construction begins with the base vector (i.e. there are no pre-existing sub-combinations already built) then the minimal number of rounds required to complete any given combination (n) can be found by: ⌈log_2_(n)⌉+1. The half braces ⌈⌉ represent the ‘ceiling’, i.e. round up to the nearest integer. Calculations show that sub-cloning is the most time-efficient construction strategy for any combination of 4 or more insertions (**Figure 6b**).

### Sub-cloning sub-combinations

**Figure 7:**
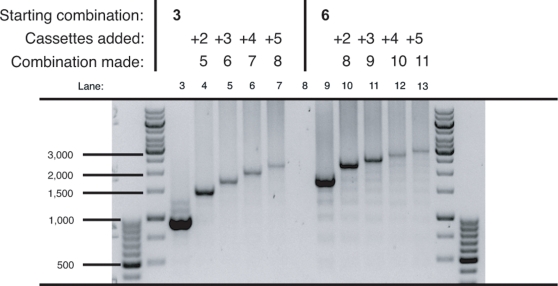
Combinations of up to eleven built from sub-cloned donor fragments. Sub-cloned donor fragments of 2–5 cassette combinations (excised with the core 2 enzymes) were inserted into both 3 and 6 cassette recipient plasmids, successfully creating new combinations of 5, 6, 7, 8 (two combinations thereof), 9, 10 and 11. Insertions were confirmed by PCR screening using primers that flanked the MCS region. Each insertion added ∼270 bp to the size of the previous recipient plasmid.

Several different combinations were assembled from component sub-combinations of increasing number of cassettes. Double, triple, quadruple and quintuple cassette fragments were excised from suitable donor plasmids using the core 2 sub-cloning enzymes, *Mlu* I and *Pac* I. Each fragment was inserted into both a 3 and 6 cassette recipient plasmid opened up with the core 2 recipient enzymes, *Asc* I and *Pac* I. The successful creation of 5, 6, 7, 8, 9, 10 and 11 cassette plasmids was confirmed by Pfu-based PCR analysis (**Figure 7**). This example showed that the sub-cloning methodology is sound and that PCR-generated and sub-cloned donor fragments (of different lengths) could be combined together. Moreover, it demonstrated that the Pfu-based PCR is capable of amplifying templates of at least 11 cassettes. It should also be noted that even though all cloning was directional, cloning success was much improved by complete digestion (typically overnight), de-phosphorylation, and purification of the recipient vectors prior to all ligations (as detailed in the methods). On average, ∼80 % of screened colonies were positive - even when working with up to 11 cassettes. Shortcutting any of these steps often resulted in an unsuitably high number of religated recipient vectors that made screening arduous, inefficient and notably increased the total construction time.

## Discussion

This work details two key solutions to problems commonly faced when working with multiple hairpin vectors: (**1**) an infinitely expandable cloning strategy based on recycling a unique set of RE recognition sites by repeatedly destroying and restoring them with every round of cloning, and (**2**) a Pfu-based PCR method capable of amplifying at least 11 repeated hairpin expression cassettes. The cloning strategy overcomes a lack of suitable recognition sites often encountered in complex cloning strategies such as those requiring repeated insertions and or large recipient vectors. The procedure is technically simple to execute as digestion conditions are repeated and therefore only optimized once. The cloning can be considered ‘ideal’, as each step is directional with a high success rate that minimized screening effort. Projects of few insertions can be made easily by consecutive rounds of insertions adding one cassette at a time into the final recipient vector. Large projects with 4 or more insertions are most efficiently made with parallel lines of construction in multiple vectors that are progressively joined. Our cloning strategy was verified by constructing combinations of up to 11 hairpin expression cassettes from both PCR and sub-cloned donor fragments, which as far as is known, is the largest reported combination (the largest outside of this study being 6) [7]. The Pfu-based PCR was a critical development for making combinations of this size since Taq-based reactions were ineffective for screening combinations of 5 or more cassettes.

Cloning success is governed by several factors. RE choice is one of these. Some enzymes are more reliable ‘cutters’ than others. These enzymes are identified by experience and manufacturer notes (e.g. NEB catalog & Technical Reference, 2007–08). Selecting enzymes with similar double digestion conditions can also facilitate the ease of cloning. It may be possible to assemble more enzyme pairs with compatible recognition sites than those considered here if using additional enzymes sourced from other suppliers. Also, extra time spent preparing the vectors (digestion, de-phosphorylation and cleanup) increased the percentage of positive colonies and thus reduced the number that needed to be screened.

As with any RE-based cloning strategy, the recognition sites used in the core cannot be present in any of the inserts. Our multiple core design can mitigate this limitation by providing alternative recognition sites for insertion. Even though the strategy conceptually allows for an infinite number of repeated cloning events, there will likely be a practical limitation to the number of events achievable in different vector and or host systems. While it's technically possible for large insert sequences (from 300–1200 kb) to be maintained in bacterial hosts [9]–[11] increased vector size leads to reduced transformation efficiency, increased metabolic burden (for the host), reduced copy number, and reduced ligation efficiencies [9], [12], [13]. It has been reported that bacterial transformation by electroporation can improve the success rate of cloning in larger vectors [9], [12], as can using a reduced amount of selectable marker in bacterial host cultures (Promega FAQspeak 0030). External shuttling capacity was specifically built into the design reported here to provide the option of working mostly within small, simple and easy to use vectors if required.

Several other strategies developed for multiple cassette cloning in different situations were reviewed and compared [7], [14]–[16]. All were single-core strategies using compatible and unique or blunt enzyme combinations in different configurations. Each has it its advantages and although elegant in their varied use of compatible enzyme pairs, they are all inherently less tolerant of donor fragments that contain core recognition sites as they are all single core designs. The multi-core strategy described herein is advantageous in providing maximum flexibility in the choice of cloning sites (both within primers and across cores), and hence compatibility with as many donor sequences as possible. We have explored the construction options in this report and found the most time-efficient solution for constructing combinations of any number. There are, however, many ways in which the described strategy could be altered. One of these would be to replace the ‘b’ and ‘B’ enzymes with a single unique recognition site (similarly to the strategies described by others), enabling twice as many cores to be simultaneously built (up to four in this example). Other possibilities include cross-core cloning by using compatible sites from different cores in multi-core designs, or even designing cores within cores.

In summary, the experiments have shown that our expandable cloning strategy is practically sound, and has the potential capacity for both PCR and sub-cloned donor fragments to be interchangeably inserted ad infinitum. The directional cloning strategy is a general method that is technically simple and can be tailored to any vector or cloning scenario, as the cores can be adapted to any suitable enzyme sets. The Pfu-based PCR method makes it possible to use PCR in complex multiple hairpin projects where Taq-based methods become unsuitable (>4 cassettes). While the solutions were devised and proven to solve the multiple shRNA problem, both are equally useful in other cloning situations using repeated sequences and or requiring more than one insertion.

## Methods

### MCS Construction

The multiple cloning site was assembled by annealing two complementary synthetic oligonucleotides (shown divided at each feature); the upper oligo (5′-3′): TCGA ACCGGT ACTAGT GCTAGC GCTAAGC TGTACA ACGCGT GGCGCGCC CCCGGG TTAATTAA AACGTT CACGCAGTG A, and the lower oligo (5′-3′): CTAGT CACTGCGTG AACGTT TTAATTAA CCCGGG GGCGCGCC ACGCGT TGTACA GCTTAGC GCTAGC ACTAGT ACCGGT. The synthetic MCS insert was designed to have overhanging ends that were complementary to *Nhe* I and *Xho* I (also *Sal* I) digested overhangs, but non-restorative on ligation. The MCS was inserted into a recipient plasmid (a 7 kb carrier plasmid encoding a lentiviral transfer vector), digested with *Nhe* I and *Xho* I. The recipient plasmid was a derivative of pKC(ro^−^)MND.MCS obtained from Cell Genesys.

### PCR cassette insertions

The individual shRNA expression plasmids used as PCR templates were constructed as part of another project using a Phi29 template extension method as previously described [17]. The core 1 PCR primers (used successfully, but not demonstrated herein) were: forward (5′-3′): GC ACTAGT **GTT TTC CCA GTC A** **CG AC**, and reverse (5′-3′): GC CGTACG TGTACA GCTAAGC GCTAGC **GCT GCA ATA AAC** **AAG TTA**. The core 2 PCR primers were: forward (5′-3′): GC ACGCGT **GTT TTC CC** **A GTC ACG AC**, and the (5′-3′): GC GCGATCGC TTAATTAA CCCGGG GGCGCGCC **GCT GCA ATA AA CAA GTT A**. Each primer consisted of a small terminal ‘seat’ (to facilitate RE binding), overhanging recognition sites to be included in the product, and the primer binding site (shown in bold). Each PCR consisted of the core 2 primers (20 pmol each), 1x PCR II buffer (Roche) 2.5 mM MgCl_2_, 10 mM dNTPs (each), ∼100 ng of template, 0.5 µl AmpliTaq-Gold (Roche), and H_2_O to a final volume of 50 µl. Each PCR was cycled at 1x: 94°C for 10 min., 35x: 94°C for 30 sec. | 55°C for 30 sec. | 72°C for 30 sec., and 1x 72°C for 10 min. End digestions (core 2) were conducted directly in the PCR mix (after cycling) by adding 5 µl of 10x BSA, 1 µl each of *Mlu* I and *Asi* SI and incubating @ 37°C for a minimum of 1 hr. All REs were sourced from New England Biolabs. Digested cassettes were separated on 2 % TAE agarose gels, gel extracted (Qiagen Gel Extraction kit) and eluted in 35 µl of H_2_O. Recipient plasmids were prepared by digestion of ∼10 µg with 1 µl each of *Asc* I and *Pac* I, NEB 4 buffer, BSA plus H_2_O to a final volume of 50 µl and incubation at 37°C overnight. This was followed by heat inactivation (65°C for 20 min.) and de-phosphorylation by adding 5 µl Antarctic Phosphatase, 5 µl buffer and incubating at 37°C for a minimum of 1 hr. Antarctic Phosphatase was heat inactivated (65°C for 10 min) prior to separating the DNA on 1 % TAE agarose gels and gel extraction (performed as already described). Single donor cassettes were ligated into the linearized recipient plasmid using 4 µl of vector, 6 µl of hairpin cassette, 10 µl of Quick DNA ligase buffer and 1 µl of Quick DNA ligase (NEB). The ligations were incubated at room temperature for 5 min., and then purified using the QIAgen PCR Purification kit by mixing with 5 volumes (105 µl) of Buffer PB, and eluting in 35 µl H_2_O. Ligated products were transformed by electroporation under standard conditions, and positive colonies were identified by a direct colony PCR technique. All plasmids were propagated in GT116 *E. Coli* cells; a cell line specifically developed for the replication of hairpin containing plasmids (Invivogen). DNA was extracted (Hi-speed Maxi-prep Kit, Qiagen) and quantitated in triplicate (Nanodrop).

### Sub-cloning

Sub-cloned donor fragments were prepared from plasmids with 1 or more PCR cassettes already inserted by digestion of ∼10 µg with 1 µl each of *Mlu* I and *Pac* I, NEB 2 buffer, BSA plus H_2_O to a final volume of 50 µl and incubation at 37°C overnight. All subsequent cloning steps were done as previously described.

### Pfu-based PCR screening and gel electrophoresis

Inserted donor fragments were screened by gel analysis of PCR amplicons made using primers that flanked the MCS; forward (5′-3′): AGT TCT GCA CTC GGC CTC TG, and reverse (5′-3′): CCA TGG TCT GCA GTC GCT AG. These were positioned 38 bp upstream and 21 bp downstream (inclusive). The optimized Pfu-based PCR screening method consisted of the primers (20 pmol each), 1x Pfu Ultra II HS buffer (Stratagene), 3.5 mM MgCl_2_ (total), 10 mM dNTPs (each), ∼10 ng of template, 2.5 µl DMSO (5 %), 0.5 µl Pfu Ultra II HS (Stratagene), and H_2_O to a final volume of 50 µl. Each PCR was cycled at 1x: 95°C for 2 min., 35x: 95°C for 20 sec. | 66°C for 20 sec. | 72°C for 0.5–4 min. (depending upon template length), and 1x 72°C for 3 min. Samples were electrophoresed on 1.7 % TAE agarose gels plus 0.01 % SyberSafe stain (Invitrogen). The Generuler 100 bp and 1 kb DNA ladders (Fermentas) were run as size markers.
